# Polysomnographic scoring of sleep bruxism events is accurate even in the absence of video recording but unreliable with EMG-only setups

**DOI:** 10.1007/s11325-019-01915-2

**Published:** 2019-08-12

**Authors:** Tomi Miettinen, Katja Myllymaa, Anu Muraja-Murro, Susanna Westeren-Punnonen, Taina Hukkanen, Juha Töyräs, Reijo Lappalainen, Esa Mervaala, Kirsi Sipilä, Sami Myllymaa

**Affiliations:** 1 Department of Applied Physics, University of Eastern Finland, PO Box 1627, FI-70211 Kuopio, Finland; 2 Institute of Dentistry, University of Eastern Finland, Kuopio, Finland; 3 Department of Clinical Neurophysiology, Diagnostic Imaging Center, Kuopio University Hospital, Kuopio, Finland; 4 Institute of Clinical Medicine, University of Eastern Finland, Kuopio, Finland; 5 Department of Oral and Maxillofacial Diseases, Kuopio University Hospital, Kuopio, Finland; 6 Oral and Maxillofacial Department, Medical Research Center Oulu, Oulu University Hospital, Oulu, Finland; 7 Research Unit of Oral Health Sciences, Faculty of Medicine, University of Oulu, Oulu, Finland

**Keywords:** Sleep bruxism, Masticatory muscle activity, Electromyography, Electroencephalography, Audio, Video

## Abstract

**Purpose:**

To determine the accuracy of scoring masticatory muscle activity (MMA) events in seven different polysomnography (PSG) setups.

**Methods:**

Nineteen volunteers (13 females, 6 males, age 31.1 ± 12.9 years, 12 self-proclaimed bruxers) attended one-night PSG recording, supplemented with audio, video, and a separate frontal electroencephalography electrode set (FES). The same examiner scored the MMA events with seven different setups, with varying number of channels available: (1) one, (2) two, and (3) four EMG channels, (4) PSG without audio or video (PSG-N), (5) home PSG with FES and audio (FES-A), (6) PSG with audio (PSG-A), and (7) PSG with audio and video (PSG-AV). A subset (*n* = 10) of recordings was scored twice to determine intra-scorer reliability. MMA indices and accuracy of scoring the events in different setups were compared against PSG-AV.

**Results:**

The intra-class correlation coefficient (ICC) between PSG-AV and PSG-A was high (0.940, *p* < 0.001) as well as for FES-A (0.927, *p* < 0.001), whereas for PSG-N, it was lower (0.835, *p* < 0.001); for setups with only EMG channels, coefficients were very low (ICC < 0.100 for all). Intra-examiner reliability was high (ICC > 0.939 for all setups), with the exception of PSG-N (ICC = 0.764, *p* = 0.002). When comparing against the MMA events scored in PSG-AV, the sensitivity of MMA event recognition for PSG-A was 78.5% and specificity 95.5%, which were substantially higher than sensitivity (52.0%) and specificity (87.2%) of PSG-N.

**Conclusions:**

MMA event scoring accuracy with PSG-A or FES-A is almost comparable to PSG-AV. Since precise event recognition is essential for accurate MMA scoring, it is evident that one cannot rely exclusively on EMG.

## Introduction

Sleep bruxism (SB) is masticatory muscle activity (MMA) that occurs involuntarily during the sleep [1]. The latest international consensus paper classifies SB as a behavior that can be a risk (or in some cases, protective [2]) factor for certain clinical consequences, such as tooth wear and transient pain sensations in the orofacial area [1, 3, 4]. A definitive SB grading can only be achieved by some kind of instrumental assessment (i.e., biosignal recordings) [1, 5, 6]. Accuracy, applicability, affordability, and accessibility are the main requirements of any feasible method for assessing SB [1]. However, these properties vary extensively between the different setups that are currently used to perform the instrumental (and also non-instrumental) assessment [5, 7].

Polysomnography with audio and video recordings (PSG-AV), where MMA events are quantified by using generally agreed scoring rules for data from electromyographic (EMG) channels of masticatory muscles, has been long considered as the gold standard for accurate SB assessment [6, 8]. The audio and video footage enable the recognition of orofacial activities (OFA) and other muscle activities (OMA) that could easily be mistaken for MMA events and thus lead to an overestimation of the severity of SB [9–12]. Accurate scoring of MMA events even for multiple nights is important for obtaining reliable results of the level of SB activity due to relatively high night-to-night variability of MMA events, which is even higher when OFA and OMA events are not excluded [13–17]. The ratio of MMA events to OFA and OMA events is highly patient-depended [10], and no significant correlation was found in the number of OFA/OMA events per hour of sleep between two consecutive nights in a group of bruxers with low MMA index [16]. Even though the PSG-AV has excellent accuracy, it is not very applicable, affordable, or even accessible. For that reason, several studies have been conducted to assess the accuracy of different, more applicable, affordable, and accessible setups which have been compared against PSG-AV [5, 7, 12, 18–20]. Carra et al. compared the PSG with and without audio and video recordings and found a 24% overestimation in the MMA index when no audio or video footage was used in scoring [12]. Several studies have reported varying agreement levels between setups that use only EMG compared to PSG-AV [7, 19, 20]. Our group has previously compared a forehead electrode set (FES) recording for PSG-AV and found that the MMA event indices recorded fully with FES correlated well with the values obtained with PSG-AV [21]. We also found that in the case of FES recordings, the exclusion of video footage and sleep stage scoring slightly reduced the accuracy of the MMA event scoring [21]. However, no direct comparison has been made between the scoring accuracy of PSG without video and PSG-AV. Furthermore, verification is needed for the results of a higher MMA scoring accuracy when sleep stage scoring is included [21] or when only the MMA events during sleep periods are taken into account in the assessment of SB activity [18]. This further verification is needed due to the fact that even though it is true that many of the OFA and OMA events (26–72%) occur during short periods of wakefulness [12, 18], so too do some of the MMA events (26%, [12]).

The main shortcoming in our current knowledge is that the accuracy results obtained in previous studies are not directly comparable with each other due to the extensive variations in measurement setups, scoring rules, and statistical analyses. We have not found experimental studies that would have compared a wide range of setups with varying numbers of channels against the gold standard PSG-AV. Arguably, this comparison is a prerequisite for conducting a comprehensive evaluation of the pros and cons of the different instrumental approaches.

The purpose of this study was to compare the manual scoring accuracy of different reduced setups to the gold standard PSG. We chose to compare six setups against PSG-AV: (1) EMG-only setup with one, (2) two or (3) four channels, (4) PSG with no audio or video (PSG-N), (5) FES with audio (FES-A), and (6) PSG with audio (PSG-A). Our working hypothesis was that the setups consisting of EEG, submental EMG, and EOG channels together with an audio signal, PSG-A, or FES-A, i.e., the PSG setups without video would have highest accuracies whereas those setups consisting of only EMG channels would be rather inaccurate.

## Methods

### Subjects

Nineteen out of the original 31 volunteers that had a successful full-night PSG recording in sleep laboratory (originally conducted for the feasibility study of the FES, see [21, 22]) were selected for this study. Subjects were excluded from this study if they had any of the following: (1) missing audio or video footage (nine subjects), (2) video footage was not focused on the facial area of the subject (two subjects), or the (3) recording had missing data on any one of the recorded channels that were used for scoring (one subject). The included subjects were 31.1 ± 12.9 years old (mean ± standard deviation), 13 were women and six men, 12 were self-proclaimed bruxers, six were not, and one was unknown. Informed consent was obtained from all individual participants included in the study.

### Recordings

All subjects went to sleep in an in-hospital sleep laboratory at their regular bedtime and were woken up in the morning at the time of their request. The PSG montage was recorded with the EMBLA N7000 system (Embla, Broomfield, CO, USA) with six EEG derivations (F4-M1, F3-M2, C4-M1, C3-M2, O2-M1, O1-M2), two electrooculographic derivations (E1-M1 and E2-M1), and chin EMG. For MMA event scoring, bipolar EMG derivations placed bilaterally on the masseter and temporal muscles were recorded. A frontal electrode set (FES) montage was recorded alongside the PSG montage consisting of the following derivations used in this study [21]: EEG: Af7-T9, Af8-T10, F7-T10, F8-T9, T10-T9; EOG: Fp1-T9, EOG-T9. Concomitant audio and video recordings were also conducted by having a video camera with a microphone in close proximity to the subject in the caudal direction (maximum distance around 1 m from the bed) with the camera being focused to record events in the facial area. For the synchronization of PSG channels and audio-video footage, we recorded all channels and audio-video footage in the same software (RemLogic, Embla). The synchronization of PSG channels and audio-video footage in each recording was verified before scoring against three maximal voluntary clenching tasks that the patient was asked to perform at the beginning and at the end of the recording. A detailed description of the entire setup can be found elsewhere [21].

### Scoring

MMA, OFA, and OMA events were scored by one scorer (T. Miettinen) in five rounds for the different PSG setups. Before any scoring rounds, all recordings in each scoring round were pseudonymized and arranged in a random order. At least 3 weeks passed between each subsequent scoring round. In the first scoring round, MMA events were scored by having only four EMG channels visible from the masseter and temporalis muscles (bilaterally). Events were scored on the following basis: on how many channels did the MMA event pattern appear: (1) if the pattern appeared on the right masseter channel, it was scored as a 1-channel MMA event, (2) if the pattern appeared on both of the masseter channels, it was scored as a 2-channel MMA event, and (3) if the event pattern appeared on at least 3 of the 4 EMG channels, it was scored as 3/4-channel MMA event. No OFA or OMA events were scored during this round.

In the following four scoring rounds, the following channels were used: EEG (from either the FES or PSG setups), submental EMG and EOG channels, and a varying number of masseter/temporalis EMG channels. In the second scoring round (PSG without audio or video, PSG-N), all four masseter/temporalis EMG channels were used for scoring the 3/4-channel MMA events. Large abrupt shifts (> 50–100 μV) in EEG, chin EMG, and/or EOG channels were used to recognize OMA events and to distinguish them from MMA events. In the third scoring round (FES and audio, FES-A), two masseter channels and EEG and EOG channels from FES montage were used (to simulate a home PSG setup with a reduced number of channels). Two-channel MMA events were scored and the audio channel was available in addition to the EEG, EOG, and EMG channels to help the scorer recognize the MMA, OFA, and OMA events. The fourth scoring round (PSG with audio, PSG-A) was similar to the second one, with the exception of the inclusion of audio recordings that were used to recognize MMA, OFA, and OMA events. In the fifth round (PSG with audio and video, PSG-AV), also video footage was used to detect the MMA, OFA, and OMA events in addition to all of the channels used in the previous round. Three weeks after the final scoring round, all five rounds of scoring were repeated in a randomly selected subset of recordings (*n* = 10) in a similar pseudonymized and randomized manner to determine the intra-scorer agreement.

Prior to the scoring of all events and sleep stages, EEG, EOG, and ECG channels were band-pass filtered (0.3–75 Hz), and EMG channels high-pass filtered (> 10 Hz). MMA events were scored by following widely used rules with the threshold for the MMA burst being two times the baseline activity (see [8, 12]). Events consisting of bursts shorter than 0.25 s were scored from any EMG channel as mandibular myoclonus and classified as OFA. In addition, the following OFA were scored based on any evidence from the audio or video footage: eye blinking, chewing (that was not MMA), coughing, lip or tongue movement, swallowing, talking, and yawning. OMA was scored whenever there were any kinds of large movements involved, such as the subject changing position. Two MMA, OFA, and OMA indices were calculated: for all scored events by dividing the number of events by the total recording time (TRT), and for sleep-time events only by dividing the number of events occurring during sleep by the total sleep time (TST). Based on EEG, sleep stages were scored separately by a different scorer (A. Muraja-Murro) according to AASM guidelines [23] in a pseudonymized and randomized order. An alternative rule for scoring the N1 sleep, originally made for patients that do not generate alpha rhythm [23], was applied in FES montage, since no occipital electrodes were present in that montage.

### Statistical analysis

Shapiro-Wilk test was used to determine whether the data was normally distributed. MMA index consistency and linearity between different scoring setups were assessed with intra-class correlation coefficient (ICC) and Spearman’s correlation coefficient, with PSG-AV setup as the gold standard. The intra-scorer reliability was determined by calculating the same correlation coefficients for the MMA indices in 10 repeated scorings by comparing them to the indices in the original scoring rounds. Bland-Altman plots were applied to investigate the agreement of the MMA indices between scoring setups. Paired samples *t* test and Wilcoxon signed-rank test were used to compare the distributions of MMA, OFA, and OMA indices between the scoring setups. The scorer’s performance in assessing the individual events with the different setups was compared to the gold standard (PSG-AV) setup by marking the events as either true positive (event was scored correctly with both setups), false positive (no scored event in the standard PSG setup), false negative (no scored event in the setup under comparison), or true negative (no scored event in either setup even though an event was scored in the corresponding EMG-only setup). These variables were used to calculate sensitivity, specificity, precision, and false positive rate for MMA events with each scoring setup. All statistical tests were performed by using SPSS software (version 21.0; SPSS, Chicago, IL, USA) and the threshold for statistical significance was set at *p* = 0.05.

## Results

The MMA indices scored with the PSG-A setup are very consistent and comparable to the indices scored with the PSG-AV setup: the ICC between PSG-A and PSG-AV was 0.940 (*p* < 0.001) for all scored MMA events during the recording (Table 1). For sleep-time events only, the ICC was even better, 0.970 (*p* < 0.001). The ICC value reflecting the similarity between FES-A and PSG-AV was almost as good, 0.927 (*p* < 0.001) for all events and 0.940 (*p* < 0.001) for sleep-time events only. These results are comparable to the excellent intra-scorer reliability in scoring MMA indices in any setup that had either audio or audio-video: the ICCs between two scoring rounds were between 0.939 (*p* < 0.001) and 0.981 (*p* < 0.001) for all events and between 0.956 and 0.986 (*p* < 0.001) for sleep-time events only (Table 2). Consistency with the PSG-AV declines if either audio or video is not available, as the PSG-N setup had a lower ICC of 0.835 (*p* < 0.001) for all events and 0.903 for sleep-time events (Table 1). Surprisingly, the intra-scorer ICC was reduced for the PSG-N setup: 0.764 (*p* < 0.001) for all events and 0.770 for sleep-time events only (Table 2). When only EMG channels were used to score MMA events, there was very poor consistency evident between the setups and the gold standard, as the ICC between scorings with any number of EMG channels and PSG-AV varied between 0.037 (*p* = 0.285) and 0.318 (*p* = 0.002) (Table 1). However, the intra-scorer reliability was excellent (ICC ≥ 0.948) in all EMG setups (Table 2). Similar to the estimates made with ICC, strong linear correlation with the MMA indices of PSG-AV setup (determined as Spearman’s correlation coefficient) was found with the PSG-A and FES-A setups, a slightly weaker correlation with PSG-N with the weakest correlations present with all EMG setups (Table 1).

**Table 1:** Intra-class correlation coefficients (ICC) and Spearman’s rank correlation coefficients for masticatory muscle activity (MMA) indices with the different sleep study setups when compared to standard PSG with audio-video recording (*n* = 19)

Sleep study setup	ICC	*p*	Spearman’s	*p*
1 channel EMG (all events)	0.085	0.068	0.564	0.012
1 channel EMG (sleep-time events only)	0.238	0.016	0.691	0.001
2 channels EMG (all events)	0.037	0.285	0.220	0.366
2 channels EMG (sleep-time events only)	0.193	0.058	0.425	0.070
3 out of 4 channels EMG (all events)	0.084	0.072	0.397	0.092
3 out of 4 channels EMG (sleep-time events only)	0.318	0.002	0.596	0.007
PSG, no audio or video (PSG-N, all events)	0.835	< 0.001	0.805	< 0.001
PSG, no audio or video (PSG-N, sleep-time events only)	0.903	< 0.001	0.872	< 0.001
FES with audio (FES-A, all events)	0.927	< 0.001	0.940	< 0.001
FES with audio (FES-A, sleep-time events only)	0.940	< 0.001	0.926	< 0.001
PSG with audio (PSG-A, all events)	0.940	< 0.001	0.899	< 0.001
PSG with audio (PSG-A, sleep-time events only)	0.970	< 0.001	0.948	< 0.001

*ICC* intra-class correlation coefficient, *MMA* masticatory muscle activity, *EMG* electromyography, *FES* forehead electrode set, *PSG* polysomnography

**Table 2:** Intra-scorer reliability results, intra-class correlation coefficient (ICC), and Spearman’s rank correlation coefficient for masticatory muscle activity (MMA) indices obtained with different sleep study setups when compared to a scoring with a similar setup (*n* = 10)

Sleep study setup	ICC	*p*	Spearman’s	*p*
1 channel EMG (all events)	0.985	< 0.001	0.964	< 0.001
1 channel EMG (sleep-time events only)	0.989	< 0.001	0.988	< 0.001
2 channels EMG (all events)	0.958	< 0.001	0.952	< 0.001
2 channels EMG (sleep-time events only)	0.953	< 0.001	0.952	< 0.001
3 out of 4 channels EMG (all events)	0.965	< 0.001	0.964	< 0.001
3 out of 4 channels EMG (sleep-time events only)	0.948	< 0.001	0.964	< 0.001
PSG, no audio or video (PSG-N, all events)	0.764	0.002	0.867	0.003
PSG, no audio or video (PSG-N, sleep-time events only)	0.770	0.002	0.976	< 0.001
FES with audio (FES-A, all events)	0.939	< 0.001	0.927	< 0.001
FES with audio (FES-A, sleep-time events only)	0.956	< 0.001	0.961	< 0.001
PSG with audio (PSG-A, all events)	0.978	< 0.001	0.976	< 0.001
PSG with audio (PSG-A, sleep-time events only)	0.986	< 0.001	0.960	< 0.001
PSG with audio and video (PSG-AV, all events)	0.981	< 0.001	0.951	< 0.001
PSG with audio and video (PSG-AV, sleep-time events only)	0.985	< 0.001	1.000	< 0.001

*ICC* intra-class correlation coefficient, *MMA* masticatory muscle activity, *EMG* electromyography, *FES* frontal electrode set, *PSG* polysomnography

**Fig. 1:**
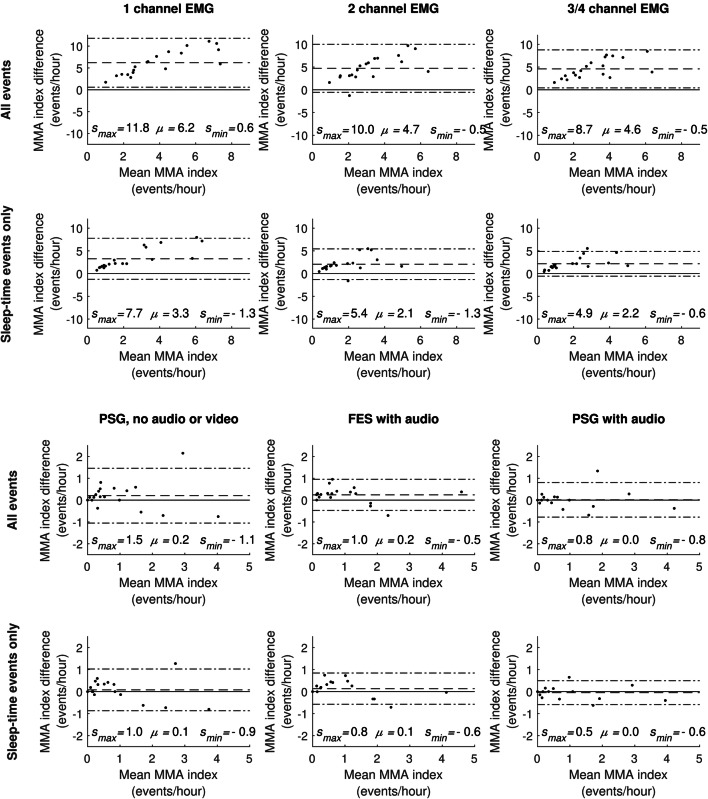
Bland-Altman plots of masticatory muscle activity (MMA) indices with the different sleep study setups. Comparison of different sleep study setups is made against polysomnography (PSG) with audio and video (PSG-AV setup). The MMA index difference is calculated by subtracting the MMA index scored with the PSG-AV setup from the MMA index of the setup under comparison. Please note that the scales of the plot axes are different for the EMG-only plots and PSG plots. *s*_*max*_: upper limit of agreement (mean + 1.96 times standard deviation of MMA difference, upper dash-dot line) *μ*: mean MMA difference (dash line), *s*_*min*_: lower limit of agreement (mean − 1.96 times standard deviation of MMA difference, lower dash-dot line)

The Bland-Altman plots (Fig. 1) show that the MMA indices scored with the PSG-A setup display excellent agreement (MMA index difference mean *μ* = 0.0 events/h and the smallest limits of agreement) with the indices scored with PSG-AV. The FES-A setup exhibited a slightly higher MMA index mean difference, but still rather good agreement. PSG-N had the MMA index difference mean at a similar level with FES-A but with substantially larger variance with respect to the difference. The agreements between the EMG setups and PSG-AV were not even close to those achieved with the other setups.

**Fig. 2:**
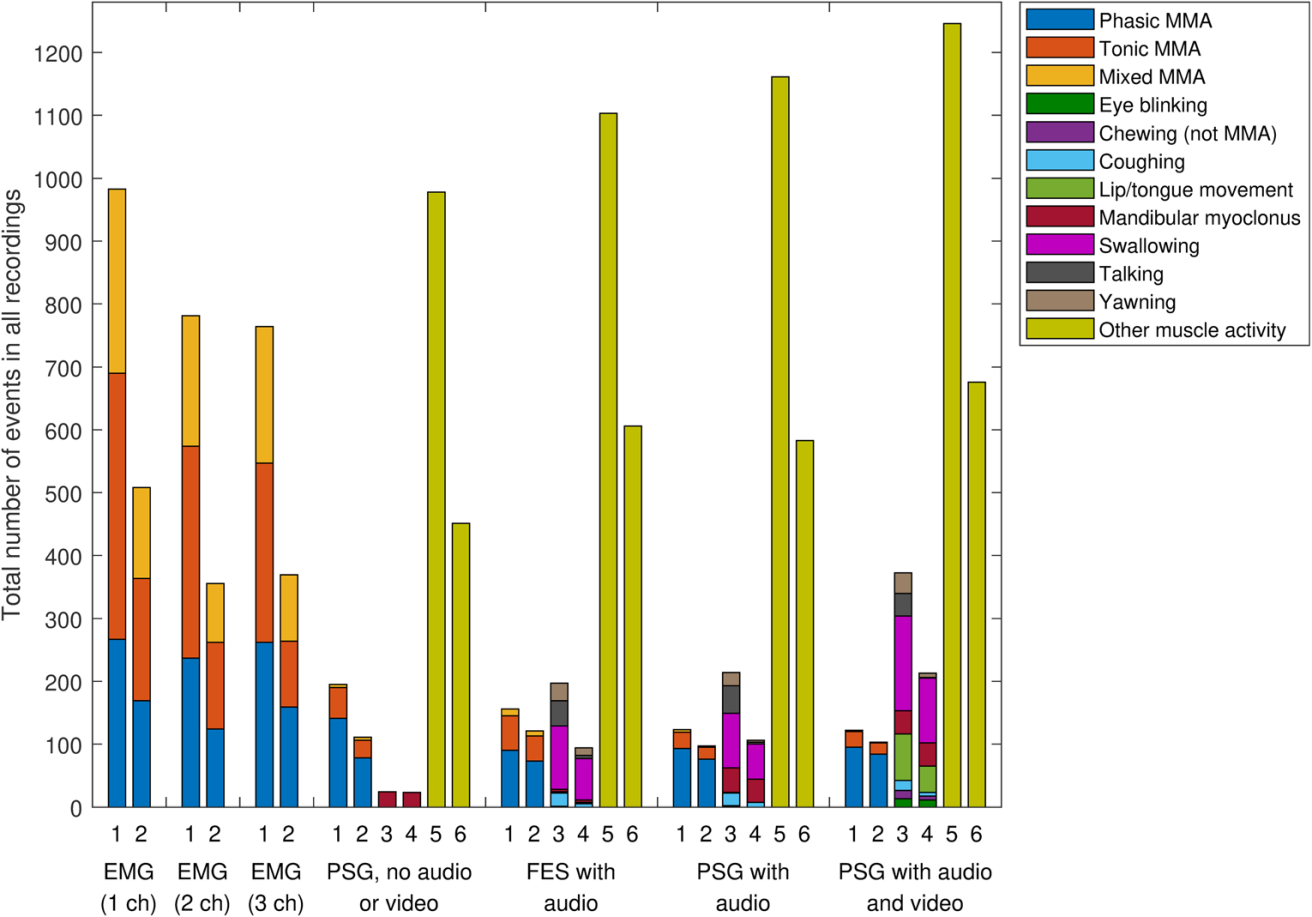
The total number of all scored events with the different sleep study setups. Data columns correspond to different event classes as follows: 1 = all masticatory muscle activity (MMA) events, 2 = sleep-time only MMA events, 3 = all orofacial activity (OFA) events, 4 = sleep-time only OFA events, 5 = all other muscle activity (OMA) events, 6 = sleep-time only OMA events

**Fig. 3:**
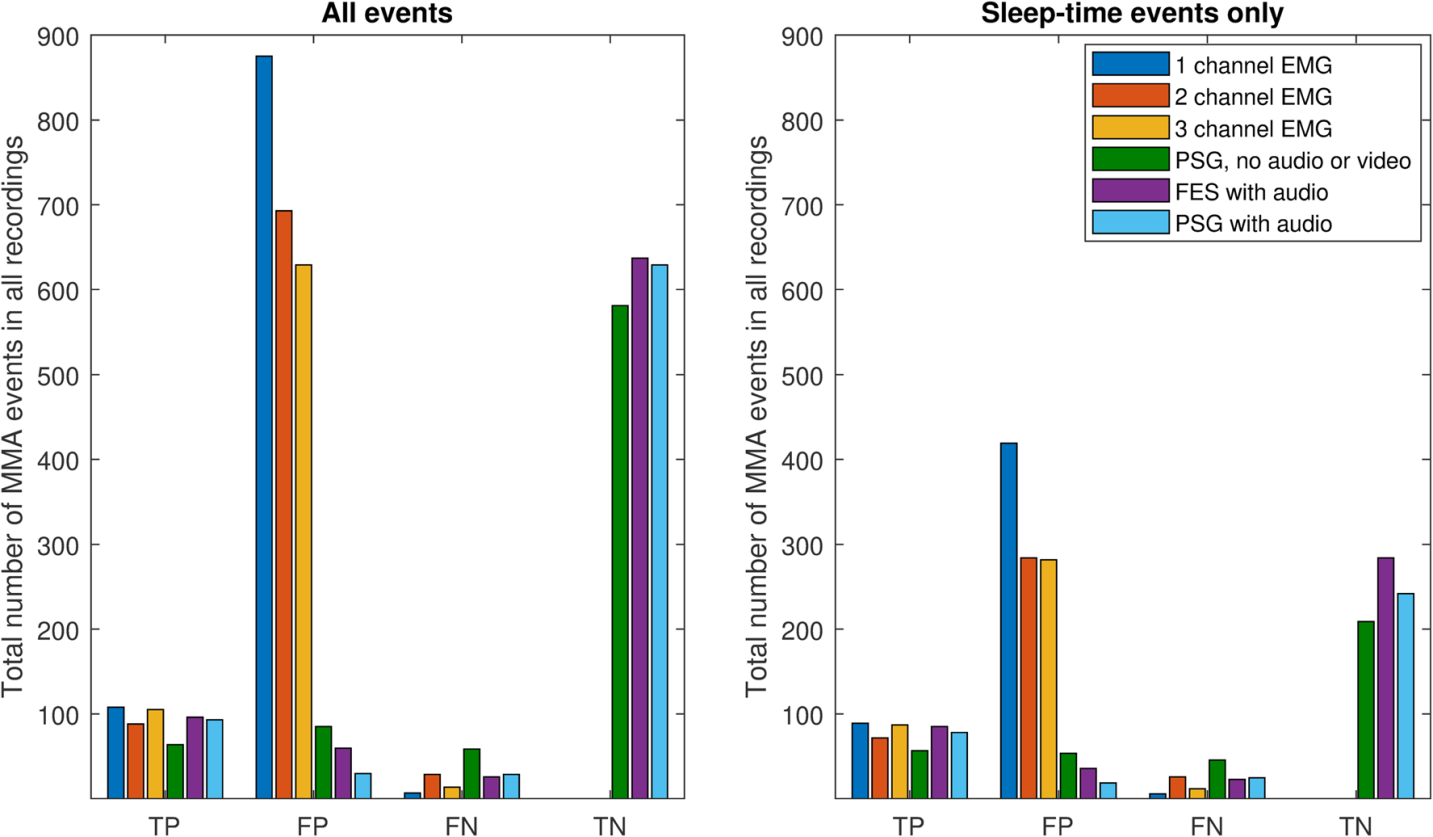
The total number of true positive (TP), false positive (FP), false negative (FN), and true negative (TN) masticatory muscle activity (MMA) events in all recordings (*n* = 19) with the different sleep study setups. The event comparison is made against polysomnography (PSG) with audio and video. *EMG* electromyography, *FES* frontal electrode set

The absolute numbers of all scored MMA events between the setups (Fig. 2) and mean MMA index comparisons (Table 3) show that significantly more events were scored with the EMG setups as compared to PSG-AV. In addition, the all-events MMA index scored with the FES-A setup was statistically significantly higher compared to PSG-AV (Table 3). As the number of scored MMA events declined, the number of OFA and OMA events increased (Fig. 2). The highest specificity, precision, and false positive rates for MMA event detection were obtained with either one of the audio setups, whereas for PSG-N setup, the values of these statistical parameters were lower (Table 4). Sensitivity was high with the 1-channel and 3/4-channel EMG setups, but precision was low with all the EMG setups. Figure 3 supports these results, as the number of false positive events in EMG setups was 6- to 20-fold higher than with any PSG setup.

**Table 3:** Masticatory muscle activity (MMA), orofacial activity (OFA), and other muscle activity (OMA) indices and statistical comparison to polysomnography (PSG) with audio and video

Variable	All events	Sleep-time events only
MMA index		
1 channel EMG	6.6 [4.0–10.3]***	3.0 [1.6–6.3]***
2 channels EMG	5.3 [3.6–8.0]***	2.2 [1.3–5.1]***
3 out of 4 channels EMG	5.4 [3.7–7.7]***	1.9 [1.5–4.8]***
PSG, no audio or video (PSG-N)	1.1 ± 0.3	0.9 ± 0.2
FES with audio (FES-A)	1.1 ± 0.2^†^	0.9 ± 0.2
PSG with audio (PSG-A)	0.9 ± 0.3	0.7 ± 0.3
PSG with audio and video (PSG-AV)	0.9 ± 0.3	0.8 ± 0.3
OFA index		
PSG, no audio or video (PSG-N)	0 [0–0.3]***	0 [0–0.3]***
FES with audio (FES-A)	1.2 [0.6–2.0]***	0.4 [0.1–0.8]**
PSG with audio (PSG-A)	1.1 [0.6–2.3]**	0.5 [0.1–1.3]**
PSG with audio and video (PSG-AV)	2.0 [1.1–4.6]	1.2 [0.4–3.1]
OMA index		
PSG, no audio or video (PSG-N)	6.0 [4.7–9.5]***	3.2 [1.6–5.0]***
FES with audio (FES-A)	6.7 [4.2–12.1]**	3.5 [2.0–8.7]*
PSG with audio (PSG-A)	8.2 [4.3–11.6]**	4.1 [1.6–6.7]***
PSG with audio and video (PSG-AV)	8.1 [5.0–12.4]	5 [2.2–8.0]

Data presented as median [25th percentile–75th percentile] (for non-normally distributed variables) or mean ± standard deviation (for normally distributed variables)

*MMA* masticatory muscle activity, *EMG* electromyography, *PSG* polysomnography, *FES* frontal electrode set, *OFA* orofacial activity, *OMA* other muscle activity

*p ≤ 0.05, **p ≤ 0.01, ***p ≤ 0.001 (Wilcoxon signed-rank test, comparison to PSG with audio and video)

^†^*p* ≤ 0.05 (paired samples *t* test, comparison to PSG with audio and video)

**Table 4:** Measures of event-wise detection performance with the different sleep study setups in comparison to standard PSG with audio and video

Sleep study setup	Sensitivity (TPR) (%)	Specificity (TNR) (%)	Precision (PPV) (%)	False positive rate (%)
1 channel EMG (all events)	93.9	–	11.0	–
1 channel EMG (sleep-time events only)	93.7	–	17.5	–
2 channels EMG (all events)	75.2	–	11.3	–
2 channels EMG (sleep-time events only)	73.5	–	20.2	–
3 out of 4 channels EMG (all events)	88.2	–	14.3	–
3 out of 4 channels EMG (sleep-time events only)	87.9	–	23.6	–
PSG, no audio or video (PSG-N, all events)	52.0	87.2	43.0	12.8
PSG, no audio or video (PSG-N, sleep-time events only)	55.3	79.5	51.4	20.5
FES with audio (FES-A, all events)	78.7	91.4	61.5	8.6
FES with audio (FES-A, sleep-time events only)	78.7	88.8	70.3	11.3
PSG with audio (PSG-A, all events)	76.2	95.5	75.6	4.6
PSG with audio (PSG-A, sleep-time events only)	75.7	92.8	80.4	7.3

Specificity and false positive rate were not calculated for EMG setups as true negatives could not be determined

*TPR* true positive rate, *TNR* true negative rate, *PPV* positive predictive value, *EMG* electromyography, *FES* forehead electrode set, *PSG* polysomnography

Variables related to sleep stage scoring show that statistically significant differences were only found between the PSG and FES setups in the following sleep variables: number of awakenings, N3 and REM sleep latencies, and sleep time in N2 and REM sleep (Table 5).

**Table 5:** Sleep variables measured with standard polysomnography (PSG) and with a frontal electrode set (FES) (*n* = 19)

Variable	PSG	FES	*p* value
TRT (min)	446.1 ± 7.0	442.5 ± 7.5	0.205^a^
TST (min)	394.9 ± 8.7	391.0 ± 10.3	0.498^a^
SE (%)	90.5 [84.3–93.8]	91.3 [82.6–95.2]	0.702^b^
WASO (min)	32.8 [18.8–53.0]	19.3 [13.0–46.8]	0.070^b^
Number of awakenings	21.7 ± 2.3	13.8 ± 1.5	< 0.001^a^
Sleep latency to			
N1 (min)	9.0 [5.4–16.4]	12.0 [6.0–22.5]	0.056^b^
N2 (min)	18.5 [13.5–35.1]	19.4 [14.5–28.4]	0.276^b^
N3 (min)	36.7 [28.9–53.6]	39.9 [31.0–56.7]	0.013^b^
REM (min)	160.0 [119.5–184.0]	220.0 [159.5–322.0]	0.005^b^
Time spent in sleep stages			
N1 (min)	46.6 ± 5.1	49.4 ± 4.6	0.553^a^
N2 (min)	225.6 ± 10.1	244.3 ± 9.9	0.017^a^
N3 (min)	73.5 [62.0–95.5]	65.0 [39.0–85.0]	0.094^b^
REM (min)	40.7 ± 3.2	26.8 ± 4.5	< 0.001^a^
W (min)	42.1 [27.7–66.0]	38.6 [18.3–59.7]	0.587^b^
Sleep stage proportion (%)			
N1	10.7 [7.3–13.8]	14.4 [6.7–17.7]	0.355^b^
N2	57.1 ± 2.1	62.7 ± 1.7	< 0.001^a^
N3	18.5 [16.9–24.1]	18.3 [11.4–21.1]	0.142^b^
REM	10.6 ± 0.9	6.8 ± 1.1	< 0.001^a^

Data presented as median [25th percentile–75th percentile] (for non-normally distributed variables) or mean ± standard deviation (for normally distributed variables)

*PSG* polysomnography, *FES* frontal electrode set, *TRT* total recording time, *TST* total sleep time, *SE* sleep efficiency, *WASO* wake after sleep onset, *REM* rapid eye movement sleep

^a^ Paired samples *t* test

^b^Wilcoxon signed-rank test

## Discussion

The present results confirm that the accuracy of MMA events scoring varies with different recording setups and this is largely attributable to the capability to recognize MMA events from OFA and OMA events. As we hypothesized, the PSG-A setup displayed the most similar MMA event scoring accuracy when compared to PSG-AV. The PSG-A setup exhibited the highest ICC and the best linear correlation with the PSG-AV, both of which were near the same level with the excellent intra-scorer accuracy. The possibility to exclude video recordings without a significant loss of scoring accuracy is especially beneficial with home PSG setups.

**Fig. 4:**
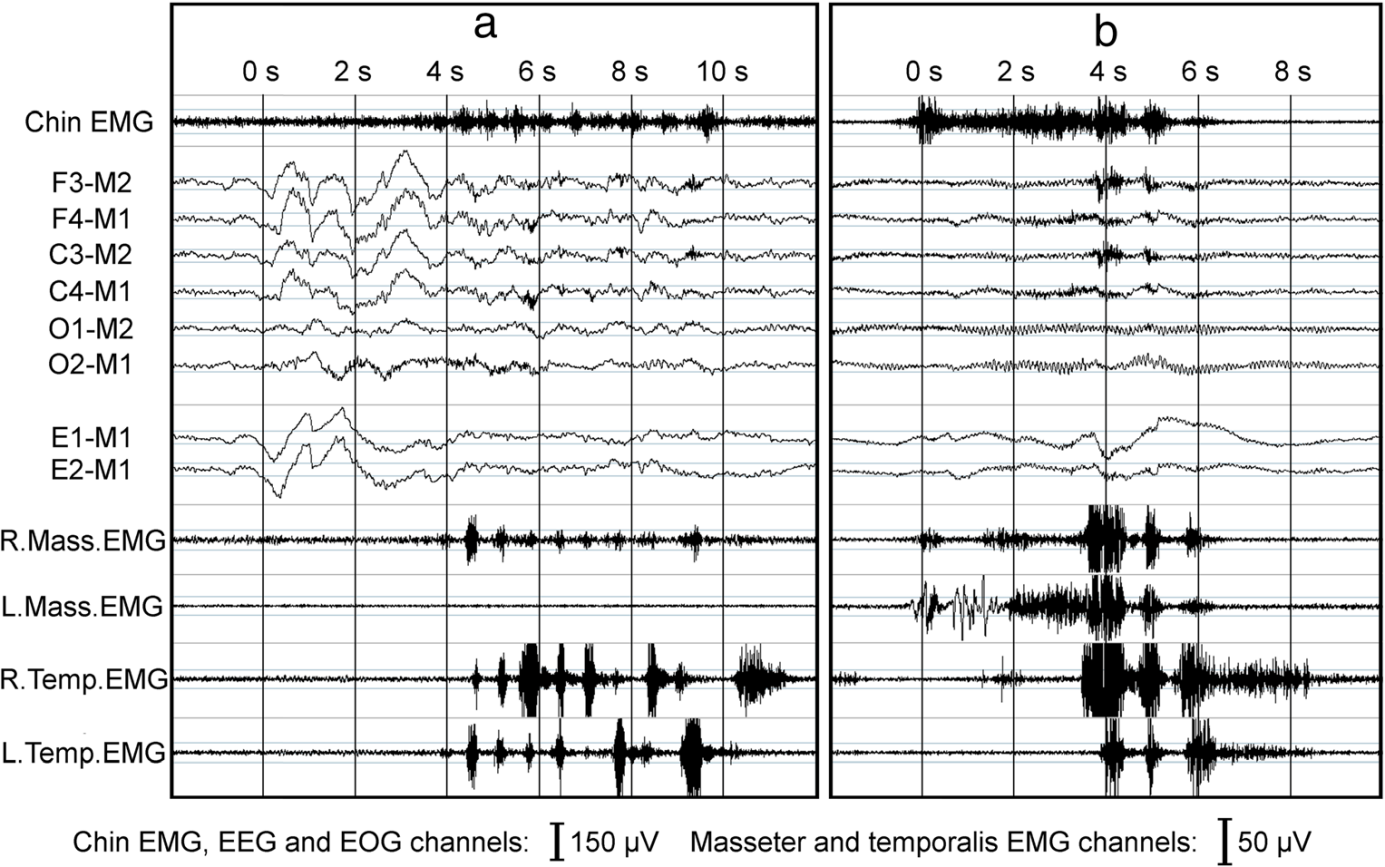
Examples of **a** missed and **b** overscored masticatory muscle activity (MMA) events in different sleep study setups utilized in this study. In example **a**, MMA event was scored in setups including (1) only right masseter electromyography (R.Mass.EMG) channel, (2) three out of four masseter or temporalis EMG channels, and (3) standard polysomnography (PSG) setup with audio and video. In PSG setups without video (with or without audio), the event was scored as other movement event, as the event in EMG occurs concomitantly with a movement-related large abrupt shift in electroencephalography (EEG) and electrooculography (EOG) channels and there were sounds of slight movement (but no tooth grinding) occurring at the same time. Furthermore, in two-channel EMG setup (consisting of R. and L.Mass.EMG channels) and forehead electrode set setup, no MMA event was scored due to no EMG activity being observed in L.Mass.EMG channel. However, video footage revealed that the subject clearly engaged in tooth grinding activity, and for this reason, the event was scored as MMA in the setup that included video footage. In example **b**, MMA event was scored in (1) all EMG-only setups and (2) PSG setup without audio and video footage. However, with audio (and video) footage, it could be determined that the activity visible in EMG channels was actually yawning. Thus, no MMA event was scored in any setup that included audio or video footage. *R.* right, *L.* left, *Mass.* masseter, *Temp.* temporalis

There seems to be two significant differences present between the accuracies of FES-A and PSG-A: on the average, more MMA events were scored with the FES-A setup and the variance in MMA index difference compared to PSG-AV was slightly higher. The differences in sleep stage scoring did not explain this discrepancy since this phenomenon was observable when events occurring during wakefulness were included in the MMA indices under comparison. The most probable explanation for the differences is the use of two EMG channels for MMA scoring in FES-A instead the of four-channel setup in PSG-A. The data in Fig. 3 reveal that more false positive events are scored with FES-A setup compared to PSG-A. It is possible that the 2-channel setups are more susceptible to detect a higher number of events (true or false), categorized here as false positives, compared to the 3/4-channel setups. This is the case also with the 2- and 3/4-channel EMG-only setups. It is concluded that there are events that are visible only on the masseter channels but not on temporalis channels. On the other hand, as the number of false negatives on 2-channel EMG setup was also higher than with the other EMG-only setups, there are also events that could easily be scored on three out of four masseter or temporalis EMG channels but not on both masseter EMG channels (Fig. 4a).

The poor accuracy of PSG-N in MMA scoring was somewhat of a surprise. There was a significantly lower correlation with PSG-AV than achieved with PSG-A and FES-A, significantly more false positive and false negative events and less true positive and true negative events, as well as a larger variance in the difference of MMA indices compared to PSG-AV. Furthermore, PSG-N suffered from poor intra-scorer accuracy (0.764), a value which was significantly lower than reported by Carra et al. for PSG-N (0.97) [12]. One possible explanation for these results could be that, with PSG-N, it is very difficult to recognize those MMA events that occur simultaneously with other body movements (16–68% of MMA events [10]). MMA could also be confused with other activities, e.g., swallowing or yawning [11, 12, 18], activities that could be easily recognized based on audio recording (Fig. 4b). Some of these other activities may have clinical relevance, especially when utilized for the recognition of other conditions besides SB (or their features), e.g., myoclonus related to rapid eye movement sleep behavior disorder [24], swallowing related to gastroesophageal reflux disorder [25], and movements related to restless legs syndrome [26]. The artifacts present in the EEG, EOG, and chin EMG channel do not appear to be very specific indicators for reliable scoring of OFA/OMA events. This is supported by the present data as the number of OFA detected was much lower with PSG-N when compared to the other PSG setups (Fig. 2, Table 3). It should be noted that we did not record leg EMG, which may be one way to improve the recognition of OMA. Carra et al. did utilize leg EMG in their study and reported higher values of ICC (0.92) between PSG-N and PSG-AV than observed here (0.835) [12].

All of the EMG-only setups had very poor accuracy. Interestingly, we found that the more channels recorded, the fewer events were scored (Fig. 1). As others have also concluded [7, 18, 20], it seems that setups based exclusively on EMG provide a poor indication of the true MMA activity. There is a significant risk for MMA event overscoring with EMG-only setups, as OFA or OMA events could not be recognized reliably (Figs. 2, 3, and 4b). Audio and video footage are considered to provide the most accurate recognition of true MMA events that involve actual tooth grinding or clenching [12]. However, it should also be noted that even with audio and video footage, scoring is not always unambiguous, and some events involving tooth grinding or clenching might go unnoticed, e.g., in cases when patient is fully under the blanket, or when true MMA-related movement blends in with other major body movements such as changing position in the video footage. The significance of these concomitant MMA and movement events for the contribution to the clinical consequences of SB is currently not clear and requires further examination. Nonetheless, it has been shown that EMG-only setups may be used for other purposes, such as determining the general level of EMG activity, e.g., by calculating root mean square for the entire signal, that has been found to be a good indicator for the occurrence of temporomandibular pain [27], but not for satisfactory MMA recognition.

Sleep stage scoring and assessing sleep-time events were found to improve the accuracy of all setups. The main reason probably for this improvement lies in the proportion of OFA/OMA events occurring during wakefulness (PSG-AV: OFA 42%, OMA 46%, calculated from data shown in Fig. 2) that is significantly higher compared than the proportion of MMA events during wakefulness (PSG-AV 12%). With respect to OMA/OFA, these percentages are lower than those reported by Yamaguchi et al. (71%) [18]. Compared to the report of Carra et al., our proportion for MMA during wakefulness is somewhat lower (12% vs 26%), for OFA somewhat higher (42% vs 26%) but for OMA, the percentages are exactly the same (46%) [12]. However, these differences may be caused by different study populations, which were rather small (*n* < 20) in all of these studies. It should be noted that the MMA events during wakefulness (just as MMA during body movements) may have clinical relevance in a similar way as the events during sleep have. In this study, the exclusion of MMA during wakefulness was only used as a means to evaluate the effect on the scoring reliability between different PSG setups, and the effect of this exclusion for the reliability of MMA index as a predictor for the clinical consequences of SB should be assessed separately.

This study has its limitations, especially due to the small study population. The original, larger study population of 31 subjects [22] had to be narrowed down to 19 subjects, as we wanted to avoid any bias on the results that would be caused by some subjects not having complete sets of scorable data or audio and video footage present in their recordings. The activities in which each subject engages during the sleep (as well as their frequency) may differ vastly [10], and the risk for bias due to individual subjects with atypical sleep behavior is higher in small study populations. In the future, it would be preferable to verify the present results in a larger population. We had only one scorer of MMA events in this study and thus cannot provide estimation of the possible inter-scorer differences with different sleep study setups. However, this issue would be interesting to assess with a wide range of setups in a similar fashion as in the present study. Furthermore, caution is advised in generalizing the findings of this study for PSG montages that differ from the present ones, as any missing or extra channels may affect the reliability of the scoring.

With manual MMA scoring, achieving high scoring accuracy unavoidably involves a certain level of inconvenience. PSG-A and FES-A were the two most accurate of the setups but also the most time-consuming to score, especially due to sleep staging and listening to the audio associated with every possible event. There seems to be a trade-off between accuracy and applicability, affordability and accessibility. A good example is that if the PSG-AV were to be replaced with the FES-A setup, one would lose only minimal accuracy away but gain significant benefits, i.e., improved applicability, affordability, and especially, accessibility due to the fact that recordings would no longer be confined to the sleep laboratory. On the other hand, for EMG-only systems, the trade-off is less favorable, i.e., improvements in applicability, affordability, and accessibility but at the cost of unsatisfactory accuracy. Besides clinical setting, accurate scoring of MMA events is also necessary for obtaining reliable results in research settings. For example, it has been proven difficult to establish links between level of MMA and the clinical findings of SB, such as tooth wear [28, 29]. As the level of MMA is highly variable between nights [13–17], and tooth wear accumulates over the course of several years, it would be beneficial to have means to study the contribution of SB in the tooth wear process accurately in the long-term follow-ups rather than with one-night studies that are commonly utilized [29]. Besides accurate scoring of MMA, this requires recordings that can be obtained in a widely available and affordable fashion and could be reliably repeated throughout the years. In the present study, we included only manually scored setups, but nevertheless, any setup, automated or manual, that is used to assess SB activity should always be tested for its event recognition accuracy and the resulting trade-offs between requirements have to be acceptable.

To conclude, accurate MMA scoring seems to be possible even without video recordings, which is especially beneficial for quantifying SB activity with home PSG. The present results showed that either audio or audio-video recordings are required if MMA scoring hopes to achieve the best accuracy; in contrast, relying exclusively on EMG is unsatisfactory and unreliable. Furthermore, it was shown that the scoring accuracy and repeatability could be improved by using only sleep-time MMA events when assessing the MMA index. Finally, it was observed that the number of EMG channels and the MMA scoring rules may affect the scoring outcome.

## Abbreviations

AASM: American Academy of Sleep Medicine
EMG: Electromyography
FES: Frontal electrode set
FES-A: Frontal electrode set with audio (setup)
ICC: Intra-class correlation coefficient
MMA: Masticatory muscle activity
OFA: Orofacial activity
OMA: Other muscular activity
PPV: Positive predictive value
PSG: Polysomnography
PSG-A: Polysomnography with audio (setup)
PSG-AV: Polysomnography with audio and video (setup)
PSG-N: Polysomnography without audio or video (setup)
REM: Rapid eye movement sleep
SE: Sleep efficiency
SB: Sleep bruxism
TNR: True negative rate
TPR: True positive rate
TST: Total sleep time
TRT: Total recording time
WASO: Wake after sleep onset
